# Receiver-Based Ad Hoc On Demand Multipath Routing Protocol for Mobile Ad Hoc Networks

**DOI:** 10.1371/journal.pone.0156670

**Published:** 2016-06-03

**Authors:** Abdulaziz Al-Nahari, Mohd Murtadha Mohamad

**Affiliations:** 1 Pervasive Computing Research Group (PCRG), Faculty of Computing, Universiti Teknologi Malaysia, Johor Bahru, Malaysia; 2 Department of Programming, Applied Science Division, Sana’a Community College, Sana’a, Yemen; Nankai University, CHINA

## Abstract

Decreasing the route rediscovery time process in reactive routing protocols is challenging in mobile ad hoc networks. Links between nodes are continuously established and broken because of the characteristics of the network. Finding multiple routes to increase the reliability is also important but requires a fast update, especially in high traffic load and high mobility where paths can be broken as well. The sender node keeps re-establishing path discovery to find new paths, which makes for long time delay. In this paper we propose an improved multipath routing protocol, called Receiver-based ad hoc on demand multipath routing protocol (RB-AOMDV), which takes advantage of the reliability of the state of the art ad hoc on demand multipath distance vector (AOMDV) protocol with less re-established discovery time. The receiver node assumes the role of discovering paths when finding data packets that have not been received after a period of time. Simulation results show the delay and delivery ratio performances are improved compared with AOMDV.

## Introduction

With the need to send and share data through networks, wireless networks provide the facility to exploit and utilize the air as a medium for transmitting data. Using such a medium makes it easier to use wireless networks in situations where there is no infrastructure for wire networks. These are called ad hoc networks. There are different types of ad hoc networks: mobile ad hoc networks, wireless sensor networks and vehicular networks. This type of network is favourable because it is an infrastructureless network which has speed and self-deployment. Ad hoc networks work for temporary periods. Their importance stems from applications such as military fields, rescue work, emergencies such as tsunamis and earthquake predictions [1]. In addition, ad hoc networks can be used for personal purposes such as sharing data, meetings and conferences. Multimedia and VoIP are also important issues that are addressed by researchers [2–4].

Mobile Ad-Hoc Networks (MANETs) consist of a set of routers (nodes) that are connected through wireless links. The absence of infrastructure in MANETs, along with different challenges such as continuous changes of network topology, limited resources in mobile nodes and application requirements to send data through multi-hop networks create real challenges to propose routing protocols in MANETs. Maintaining available routes is one of the most important challenges faced when designing routing protocols [5, 6].

Designing routing protocols that maintain routes frequently, such as in destination sequence distance-vector routing protocol, DSDV[7], and optimized link-state routing protocol OLSR [8], introduce routing protocols with substantial overheads for rebuilding a route. Thus, designing a routing protocol for real time application needs that adapts quickly to dynamic changes is preferable. Such a protocol is the well-known reactive routing protocol Ad hoc on demand distance vector routing protocol, AODV [9]. The AODV protocol selects a route from sender node to receiver node when requested. It is widely used in mobile ad hoc networks and researchers enhance the AODV algorithm depending on different criteria or challenges as in [6, 10–13]. Different comparison studies of routing protocols [14] [15] show how AODV is preferable in MANETs with different scenarios compared with standard MANET routing protocols. The challenge for improving an on-demand routing protocol is improving the maintenance phase. The maintenance phase is required when a link error occurs in the explored route; a route error packet (RERR) will be sent to all upstream senders which are using this broken link for sending data. Sender nodes re-establish the discovery phase to find a fresh valid route. This will need more time delay to find a new path for sending data packets that, in addition, affect the network throughput. The dynamic changes in network topology must be considered to improve the network performance. There are different studies on network topology properties such as [16, 17]. One of the main challenges is network reliability.

To increase the reliability of AODV protocol, a multipath routing protocol has been proposed. Ad hoc on demand multipath distance vector, AOMDV [13] works by allowing the sender node to keep multiple routes connected to the receiver node. The multipath is constructed by receiving multiple replies from the receiver node issued for each disjoint RREQ received. When an active route is broken, the sender node sends data packets through an alternative valid route; otherwise the discovery phase is re-established again.

AODV and AOMDV routing protocols take longer to re-establish new routes in high dynamic topology due to high nodes mobility. The main issue is the time delay to construct the new routes, in addition to the high overheads caused by the broadcasting of RREQ packets. In AOMDV protocol, sending data packets could suffer when these are sent through stale paths. Control overhead and packet loss would be affected in aggressive situations and multiple paths would not serve well. Different multipath routing protocols try to overcome the use of stale paths by adding probe packets through the alternative paths to check for path validity. Different enhancements to decrease the stale alternative paths have been proposed [18]-[19]. Those protocols are based on adding control packets broadcast periodically through the network, even if the sender node did not receive any error from the current path to update the alternative paths with fresh valid paths. With AODV protocol, there are other perspectives to decrease the time for finding new paths by making the receiver node broadcast RREQ packets to the sender. Huang and Chang [5] proposed a single path bidirectional route repair (BRRM) mechanism by allowing the receiver node to receive the error packet like a sender packet when link connectivity error occurs. Sender and receiver nodes broadcast discover controls with hop count equal to half the last known hop count. Intermediate nodes which hear both requests will send a reply to the sender node with information about what was received from the receiver nodes. This repair mechanism is proposed to decrease route construction time.

Another mechanism has been proposed in [20], called Enhanced receiver-based ad hoc on demand distance vector (ERB-AODV) routing protocol. This protocol reverses the discovery phase to be done by the receiver node to decrease the processing time to establish a new path. In this paper, we propose a receiver-based ad hoc on demand multipath distance vector (RB_AOMDV) which applies the single path mechanism to allow the sender node to keep alternative paths in its routing table, which in turn helps decrease the establishment of new route times. In addition, the proposed protocol increases the network reliability which is presented in multipath protocols and decreases stale path problems in which multipath protocols suffer high mobility and traffic load.

The paper structure is as follows. In Section 2, the related operations are discussed. Section 3 explains the RB-AOMDV protocol with its impact on decreasing delay time. Section 4 describes the simulation environment and parameters and shows the results. Finally, Section 5 concludes the paper.

## Related Operations

In AOMDV [13], the authors have proposed a multipath routing protocol based on AODV. The idea is to change the route discovery process by letting the receiver reply to each route request packet (RREQ), with the sender queuing the different replies as alternative paths. The authors have shown that the protocol is loop-free. In addition, the protocol assures that the routes are disjointed, there are no common between routes in links called link-disjoint paths. When the protocol is link-disjoint, each node will keep a list of alternate links that could be used if the active link is broken without alerting the sender about that error. However, an empty list means the intermediate node will send to the upstream node announcing the breakage of that link. When the sender has heard the error, the route will be deleted. The next alternate path, if one is still available, will be used as a primary path; otherwise, the discovery process will start over. The drawback of this protocol is this: what happens if the intermediate node selected an alternative path from the list that does not lead to the destination node? That means that the recovered path will be an untrusted path. Moreover, if the active route, which is called the primary path, becomes stale, the sender will select one of the alternative paths in the queue, but this alternative path could be stale as well. If all the paths became non-functioning, the sender will start the discovery of multiple routes again, which implies a long time delay.

The authors in [18] have proposed a Mobility Prediction AOMDV (MP-AOMDV) to overcome AOMDV limitations by adding a stability mechanism to the routes. The route will be chosen depending on its stability, which is calculated through the periodic heartbeat packets along the primary and alternate paths, collecting the signal strength of each path. The route with best signal strength will be chosen as the primary route. The authors [21] have proposed AOMDV-BU protocol. It is stated that AOMDV should add a backup solution. The protocol is designed in a way that when there is just one active path, the source will initiate a route discovery and find new updated paths, while the sender is still sending the data through the primary path. This discovery will happen even before the current path becomes an invalid path to create fresh alternative paths.

Another multipath solution tries to make the receiver node along with the sender node broadcast control packets to explore new paths, such as proposed protocols that are based on swarm intelligence and ant colony optimization (ACO) [19]. In addition to finding multiple paths, AntHocNet will periodically broadcast proactive ants to check the valid distribution of the paths and update the sender node using backward ants issued by the receiver node travelling to upstream nodes calculating the distribution of nodes, and the sender node updates the routing table according to best path distribution weight in [22] and [23]. The proposed protocols add QoS metrics during path exploration such as energy, delay and bandwidth. Although these protocols decrease the time needed to explore new paths using the periodic exploring mechanism even though no error occurred, high congestion and mobility are a problem these protocols may suffer from, especially with the flooding of control packets in the networks. A more reliable ant-based protocol has been proposed in [24]. Amin et al. proposed SMART protocol which makes data packets explore ways to the receiver node by themselves. The mechanism uses a swarm learning approach to decrease convergence time by using smart data packets. The learnt information can be used and updated from successive data packets and distributed though the nodes in the network in order to maintain better routes. Prabha and Ramaraja [25] proposed improved AOMDV based on link availability, neighbour node’s queuing delay node mobility and bit error rate to create multiple paths using BAT optimization. This protocol aims to decrease the discovery process time needed when current paths become broken. In [26] and [27], the authors proposed a new strategy to decrease delay and balance the load of the network by proposing a node disjoint least interference multipath routing protocol. The destination node, after replying for the first RREQ packet, waits for a period of time to receive more request packets. It then sorts the paths according to most stability paths, least time delay paths, or number of hops and replies to the source node with the list with all paths with their interferences. The source node sends data through the first main route. After receiving the list from the destination, it selects another path to send data; this path should be the least interference path with the main route. The protocol is more suitable with large network size, where there is greater ability to find different paths with least interference. With small network size, it becomes difficult to find routes with least interference nodes, and that depends on the network area size. The maintenance phase needs more consideration in order to decrease performance failure.

In [28] the authors stated the problem of time delay taken to find new routes. They proposed a QoS node-disjoint multipath protocol where the receiver node can receive multiple replies that came from the sender node during a period of time or from intermediate nodes. The receiver node calculates the bandwidth and sends a reply packet for the best estimated QoS path. Other reply packets are sent back to the sender depending on the quality of the path and number of hops. This protocol overcomes the single path protocols but does not address the stale path problem in back-up paths or congestion situations caused by high traffic load that may affect the bandwidth as well. In [29] the authors stated the problem of congestion that increases the time delay of the network and decreases the throughput. They proposed a protocol that calculates the roundtrip time of each discovered node-disjoint path between source and destination nodes and distributes data packets along the paths using Fibonacci sequence numbers. Distributing data over paths decreases the load on nodes and decreases congestion, especially in the middle of the network. Alghamdi in [30] proposed a load balanced AOMDV routing protocol. The author takes into account balancing the energy of nodes in the network to increase network lifetime of all available multiple paths. Bandwidth of the links between nodes in each path is also considered, to decrease the problem of congested links in the network. The sender node prepares a list of qualified paths depending on criteria, energy and bandwidth, and distributes data packets through all of these qualified paths. When all qualified paths become invalid paths, the discovery phase begins all over again to explore new multiple paths with the same conditions. QoS Multipath routing protocols concerned with energy, load balancing, delay or bandwidth such as [31], [32], [33], [34] and [35] use the receiver node to wait for different replies and to make decisions based on QoS constraints collected by the request packets, then send the reply packet back to the sender node. On the other hand, to send a reply packet for each requested packet received and during the journey of the reply packet, the QoS constraints are calculated by intermediate nodes until the sender node receives all replies and then decides which path is better to use and which paths are alternatives. In both situations, more time is needed to do the set-up of a path which increases the time delay for sending data packets successfully to the receiver node.

ERB-AODV routing protocol reverses the discovery phase to be done by the receiver node to decrease the set-up time of a path during communication. After receiving the first RREQ packet from the sender node, the receiver node broadcasts a receiver RREQ packet to the sender node, and starts the waiting time to check whether data are received. If data are not received, the receiver RREQ packet is rebroadcast and a period of time is spent waiting; this process is repeated for a fixed number of retrials, which is the same mechanism used by the sender node when waiting to hear a reply packet from the receiver node. Increasing reliability is an important factor, and that is achieved by employing single path protocol to work as multipath protocol. By taking into account aggressive scenarios such as high congestion, multipath protocol has a problem with end to end delay. Delay is increased because during communication time, alternative paths could be stale paths or busy paths with high loaded memory. Re-establishing new multiple paths with less time is another challenging issue when designing a multipath routing protocol. Therefore we employed the ERB-AODV protocol to work as multipath as AOMDV protocol by proposing RB-AOMDV protocol to take its fast path discovery, adding the advantage of reliability in sending through multiple paths.

## Receiver-Based Ad Hoc On Demand Multipath Distance Vector Routing Protocol

In this section, we discuss the improvement to the AOMDV routing protocol by employing the receiver node to update the sender node with new paths. The improved routing protocol is called receiver-based ad hoc on demand multipath distance vector routing protocol (RB-AOMDV). This routing protocol is a node disjoint multipath routing protocol where sender node and receiver nodes explore multiple node-disjointed paths, reverse paths and forward paths respectively. The receiver nodes play different roles compared with AOMDV. It will not just send a reply packet to each disjoint request packet, it will also broadcast reverse request packets to the sender node when detecting that no data have been received. This mechanism reduces the set-up operation needed when changes in topology occur through high mobility, or there is high load in the network because of high data traffic (e.g. high collision or memory overload). In AOMDV, each intermediate node sends a single hop HELLO packet to update the neighbour table. When data are received to be forwarded to a next neighbour node, and this neighbour is not in the neighbour table any more (i.e. the next node did not reply to the HELLO packet), then the intermediate node sends error packets to all existing sender nodes relying on the next node. Upon receiving the error packet, sender node checks the routing table for an alternative path to send data packets; otherwise it starts a new discovery process. Alternative paths can become stale paths during this time, which will cause an increase in delay and decrease the network throughput. Decreasing the time needed to find available paths is an important issue to help increase the performance of the ad hoc network. For that, RB-AOMDV routing protocol is proposed to help the sender node send data packets through a recently available path by trying to decrease the time needed to restart a discovery process from the sender node to the receiver node.

In the next subsections, we first explain how the time is reduced to set up new paths. After that, we explain the RB-AOMDV routing protocol.

### Timing analysis of the proposed protocol

**Fig 1:**
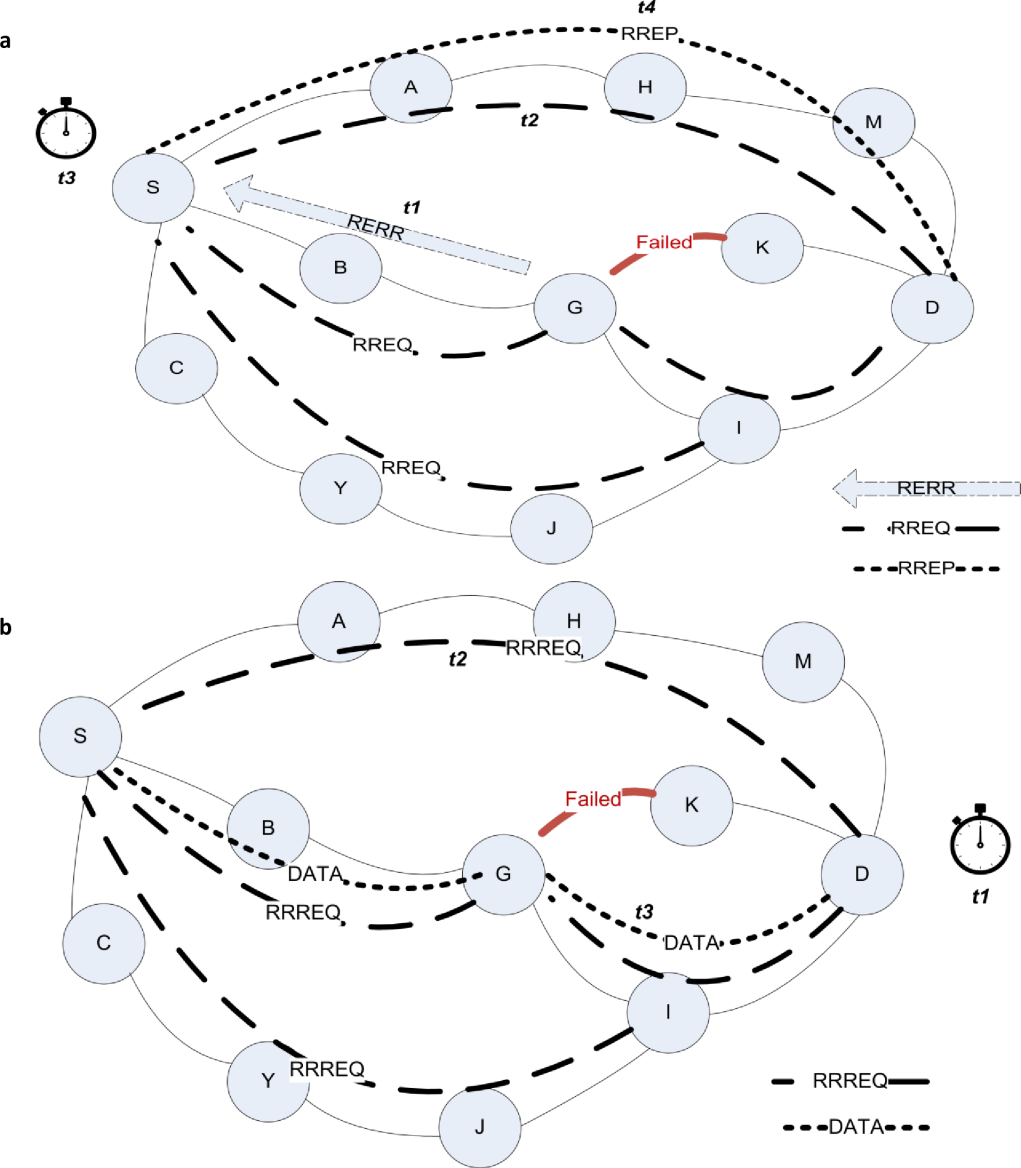
The maintenance phase in ad hoc on-demand protocol. **(a)** The ordinary reactive process when problem discovered by intermediate node, **(b)** the enhanced proposed process when problem expected by receiver node.

From Fig 1(A), let us assume that after discovery phase, the first path from source node S and destination node D is S-B-G-K-D. During the transmission of a data packet through this path, node G found node K is not feasible. Node G sends a RERR packet to inform S about the error and to any active path using K as a next path through it. Let us assume the time needed for this packet to reach S takes (t1). Upon S receiving the RERR packet, it will check the routing table and find that node A is the next alternative saved path. So the new path is S-A-E-H-M-D. If this path is fine, it means that no intermediate node is out of energy or not active because of limited resources or even moved away, and data will be sent correctly. Alternative paths’ lifetime is less than the first selected path; therefore the error can occur soon and at this point all the paths can be stale paths and S should re-establish a discovery phase.

Assuming the discovery phase takes (t2) for requested control packets to reach D, after broadcasting the requested packet, S will wait (t3) to receive the reply from D, or it tries one more time by broadcasting another RREQ packet. Finally D sends a single reply packet to S and this packet will travel in (t4) of time unit. The general time delay from this operation is formulized as follows:

Delay1=t1+n(t2+t3)+t4 (1)

where n is the number of trials to broadcast RREQ packet. The variable t3 is formulized as:

t3=x+ttl+NODE_TRAVERSAL_TIME (2)

NODE_TRAVERSAL_TIME is the time needed to send a packet from node to another node.

In RB_AOMDV, as depicted in Fig 1(B), the destination node D starts waiting for action after the first reply packet which is (t1), which is equivalent to t3 explained before. If this time expires without receiving any data, even through any of the multiple paths from the source node S, then D broadcasts a RRREQ packet looking for a new path which may take time as (t2) and waiting to receive data. Upon S receiving the packet, it sends data packets directly to the new path and the time needed for the packet to travel to the destination node is (t3). S will save all the distinct paths as new alternatives to be used. The general time delay for this operation is formulized as follows:

Delay2=n(t1+t2)+t3 (3)

When comparing the time needed between sending data packets with maintenance of routes during the transmission session, the proposed RB-AOMDV needs less time to find a new active path. Moreover, in RB-AOMDV protocol, the delay is waiting for data packets, not for reply packet as in ordinary reactive routing protocols, which decreases the delay of finding new paths and delivering data packets.

### Discovery phase

As a reactive protocol, when a source node S has data it will check the routing table for an available path to destination node D. If there is no path, S starts a discovery process to find different paths connecting it with D. S broadcasts a route request packet (RREQ) to all of its transmission range neighbours. This packet contains information of the source node, the broadcast number to prevent looping paths, destination ID and last known sequence number. Upon receiving the RREQ packet, the neighbour nodes (called intermediate nodes) check for valid active path to the destination and send reply packet to S with last known information about D. Otherwise, intermediate nodes update their routing table and build the reverse path by saving the source node and the first hop to ensure that the path is a node disjoint path. If the node received a duplicate RREQ with better metrics (here, number of hops), it just updates the routing table without retransmitting the same RREQ packet. When destination node D receives the RREQ packet, if this is not the first RREQ packet received by checking the broadcast ID, it will drop the packet. In destination node, there are three control indicators: CONFIRMED, NumofConfirm and is_data_received. NumofConfirm indicates the number of different broadcast reverse request (RRREQ) packets. The broadcast will stop when reaching a maximum threshold value while no data packets are received from the sender. This mechanism is the same as the maximum number of trials of sending RREQ packet by the sender node without hearing any RREP packet from the receiver node in AODV protocol. Is_data_received is checked to indicate whether data have been received by the receiver node. The check is performed periodically when a waiting time (Wtime) expires. Wtime is measured as the following equation:

Wtime=x+ttl+NODE_TRAVERSAL_TIME (4)

where x is a variable that indicates how long we need to hear data packets from the sender since the RRREQ has been broadcast. The ttl value is the number of hops in a path from sender to the receiver and NODE_TRAVERSAL_TIME is the time required for roundtrip transmission between two neighbour nodes. CONFIRMED is used to indicate whether the receiver is waiting to receive data or not (1 if waiting, 0 if idle).

Using these parameters, when the receiver node receives the RREQ packet from the sender node, if CONFIRMED value is zero this means there is no previous broadcast and the receiver is not waiting to hear data from the sender node. In this case, the receiver node broadcasts a reverse route request (RRREQ) packet looking for valid node-disjoint paths to the sender node and starts waiting to hear a data packet. After broadcasting RRREQ, it sets the CONFIRMED flag and resets the NumofCONFIRM counter to zero, as explained in Algorithm 1 in Box 1. The information of the packet is the same as that of the RREQ packet but the destination node here is the source node. If the receiver node receives another copy of the RREQ packet, if CONFIRMED value was one, it just drops the packet and performs no further action, unlike AOMDV where the receiver node replies to each copy of disjointed RREQ packet. There is a situation where a new RREQ packet with new broadcast ID was received while the CONFIRMED value was one. When this occurred, the receiver node replies as a unicast reply packet (RREP) for this request to rapidly discover the path. The intermediate nodes which receive the RRREQ packet only maintain a disjoint forward path and rebroadcast one copy of the packet. If a better path is found after receiving another copy of the same RRREQ packet, intermediate nodes just update the table without rebroadcasting the packet to upstream nodes. If a RRREQ packet is received from a different next hop node to the receiver node, the node inserts the information of the path in the forwarding path table, and then it will rebroadcast the RRREQ packet to its neighbours. Every time the node receives the RRREQ packet, it will check whether there are buffered data packets waiting to be forwarded to this destination. The data packets are buffered in intermediate nodes when the node is waiting for its next node to receive these data or the node was waiting for the route to be repaired. Upon receipt of a RRREQ packet by the source node, it directly sends data packets through the first discovered path. Alternative paths are saved in the routing table to be used in case of error, as explained in Algorithm 2 in Box 2.

**Box 1. Algorithm 1: RB-AOMDV receive RREQ discovery phase.**

**If** *(routetable_seq_no < rq_src_seq_no)* **then**

    *routetable_seq_no = rq_src_seq_no*

    *advertised_hops = ∞*

    *delete_table(S)*

    *insert_reverse_path(S)*

**else if***(routetable_seq_no = rq_src_seq_no)* **and** *(advertised_hops > rq_hopcount)* **then**

    *update_reverse_path(S)*

endif

**if** *(Path(i, S) = UP)* **then**

    **if** *(data_buffered(S))* **then**

        *send_data(S)*

    endif

endif

**if** *(destination)* **then**

    *dest_seq_num + = 1*

    **if** *(CONFIRMED = 0)* **then**

        *NumofCONFIRM = 0*

        *CONFIRM = 1*

        *sendRRREQ(S)*

    endif

else

    *update_RREQ(D)*

    *forward_RREQ(D)*

endif

**Box 2. Algorithm 2: RB-AOMDV receive RRREQ process.**

**If** *(routetable_seq_no < rrq_dest_seq_no)* **then**

    *routetable_seq_no = rrq_dest_seq_no*

    *advertised_hops = ∞*

    *delete_table(D)*

    *insert_forward_path(D)*

**else if** *(routetable_seq_no = rrq_dest_seq_no)* and *(advertised_hops > rrq_hopcount)* **then**

    *update_ forward _path(S)*

endif

**if** *(Path(i, D) = UP)* **then**

    if *(data_buffered(D))* **then**

        *send_data(D)*

    endif

endif

**if** *(i! = S)* **then**

    *update_RRREQ(S)*

    *forward_RRREQ(S)*

endif

### Maintenance Phase

As mentioned in the discovery phase, the receiver node sends the RRREQ packet and starts waiting time (see Algorithm 3 in Box 3). If this time expires, the node checks the_data_received value. A value of 1 means data have been received and the node re-establishes the waiting time again by resetting the data_received value. If during the waiting time no data are received, the NumofConfirm is incremented by 1 and the receiver node broadcasts new RRREQ packets with new broadcast ID and waits to hear data from the sender node. If no data are received after maximum number of sequential broadcasting RRREQ packet threshold (i.e. NumofConfirm reached the maximum threshold value), the receiver node stops waiting to hear data packets and broadcasts RRREQ packets. Algorithm 4 in Box 4 explains the receiver node action when receiving data packets.

**Box 3. Algorithm 3: RB-AOMDV send RRREQ.**

Initialize_packet(D,S)

**If** *(data_received = 1)* **then**

    *data_received = 0*

    *start_waiting_timer()*

**else If** *(NumofCONFIRM < threshold)* **then**

    *NumofCONFIRM + = 1*

    *data_received = 1*

    *send_RRREQ(S)*

    *start_waiting_timer()*

endif

**Box 4. Algorithm 4: RB-AOMDV receiving Data packet by destination node.**

**If** *(packet_type = Data and destination)* **then**

    *data_received = 1*

    *NumofCONFIRM = 0*

    *CONFIRMED = 0;*

Endif

Like the AOMDV protocol, if an intermediate node finds a problem in the link to the next node while sending data packets to the receiver node and if the number of hops to the receiver node is small, then the node will start a repair process by broadcasting RREQ packets to the receiver node and wait to hear a reply from the receiver node for a period of time called RREP_RAIT_TIME. If the node receives a RRREQ from the receiver node, it sends data packets directly through the new path, otherwise it sends route error packets (RERR) through reverse path to the sender node. The sender node removes the invalid path and checks its routing table for an alternative path and sends data packets. Although the routing table of the sender node has alternative paths not used yet, if the sender node receives a new RRREQ packet from the receiver node, it will delete all old paths and update the routing table with the new paths. This helps to make the sender nodes use the most updated valid paths and decreases the chances of using stale paths. The receiver node sends the RRREQ packet when predicting there is a problem in previous paths and there is a need to update the sender. This is because the receiver node did not receive any data during the waiting time. The sender node in some cases did not receive the error packet, especially if the intermediate nodes cannot send the error packets during high traffic load or high mobility. This situation makes the sender node keep sending data packets through the invalid path. In that case, when the receiver node finds that no data packet has been received, it should update the sender node with a currently available path.

## Implementation and Simulation Results

The proposed protocol was evaluated with AODV, AOMDV and RB-AODV routing protocols. AODV and AOMDV are well-known routing protocols in reactive protocols for MANETs. To test the performance of the proposed protocol, the results were generated using NS-2.34 as simulation software. NS-2 provides some utilities to generate random node positions with random movements depending on random-way point mobility model. The nodes are moved after each pause time in seconds to a random position with varying speed. In addition, a constant bit rate (CBR) generation has been used to generate different data rate speed, size and seed. After creating different scenarios, we ran the simulation and collected results for each protocol many times with each scenario.

### Environments

We have tested the protocols under two different scenarios. We aim to test the protocol in aggressive environments to prove how the proposed protocol is more efficient in difficult challenges such as high traffic load, high mobility or high number of nodes.

In the first scenario, the number of nodes was 32, randomly distributed in an area of 600*600 m^2^. The node max speed is 10 m/s with 0 second pause time, which makes nodes keep moving with speed variance of less than 10 m/sec. As a result, the topology changes moderately during 400 seconds of simulation time. The traffic load is varied using CBR transmission rates: 40, 80 and 100 packets per second. Packet size is fixed in all scenarios at 512 bytes. Maximum number of connections is set to be 12 sessions with different senders and receivers.

The second scenario is to test the scalability of the proposed protocol with a different number of nodes. The numbers of nodes are 40, 60, 80 and 100 nodes distributed in an area of 850*850 m^2^. Simulation time is 400 seconds. Data traffic is CBR with 512 bytes packet size, 8 packets per second transmission rate and a maximum of 12 different connections. The mobility model is the same set-up used in first scenario. Table 1 shows the simulation parameters used in this paper.

**Table 1:** Simulation parameters.

Area size	850*850 m^2^–600*600 m^2^
MAC layer protocol	IEEE 802.11
Transmission range	250m
Mobility model	Random Way Point (RWP)
Maximum speed	10 m/s
Pause time	0 sec
Traffic generator	CBR
Packet size	512 B

The RB-AOMDV protocol has been evaluated with AODV, AOMDV and RB-AODV protocols. The evaluation metrics studied are as follows:

1. Average Packet Deliver Ratio (PDR) is defined as the ratio of all data packets received to all data packets sent.
  $$PDR=\;\frac{\sum DataPacketsReceived}{\sum DataPacketsSent}$$ (5)
2. Average End to End delay (E2E) is defined as the average time of transferring data packets, since buffered by the sender node until received successfully by the receiver node.
3. Normalized Routing Load (NRL) is defined as the total of control packets sent during simulation run-time to the total of data packets transmitted successfully.
  $$NRL=\frac{TotalControlBytesSent}{Total\text{ReceivedDataBytes}}$$ (6)

### Results and discussion

**Fig 2:**
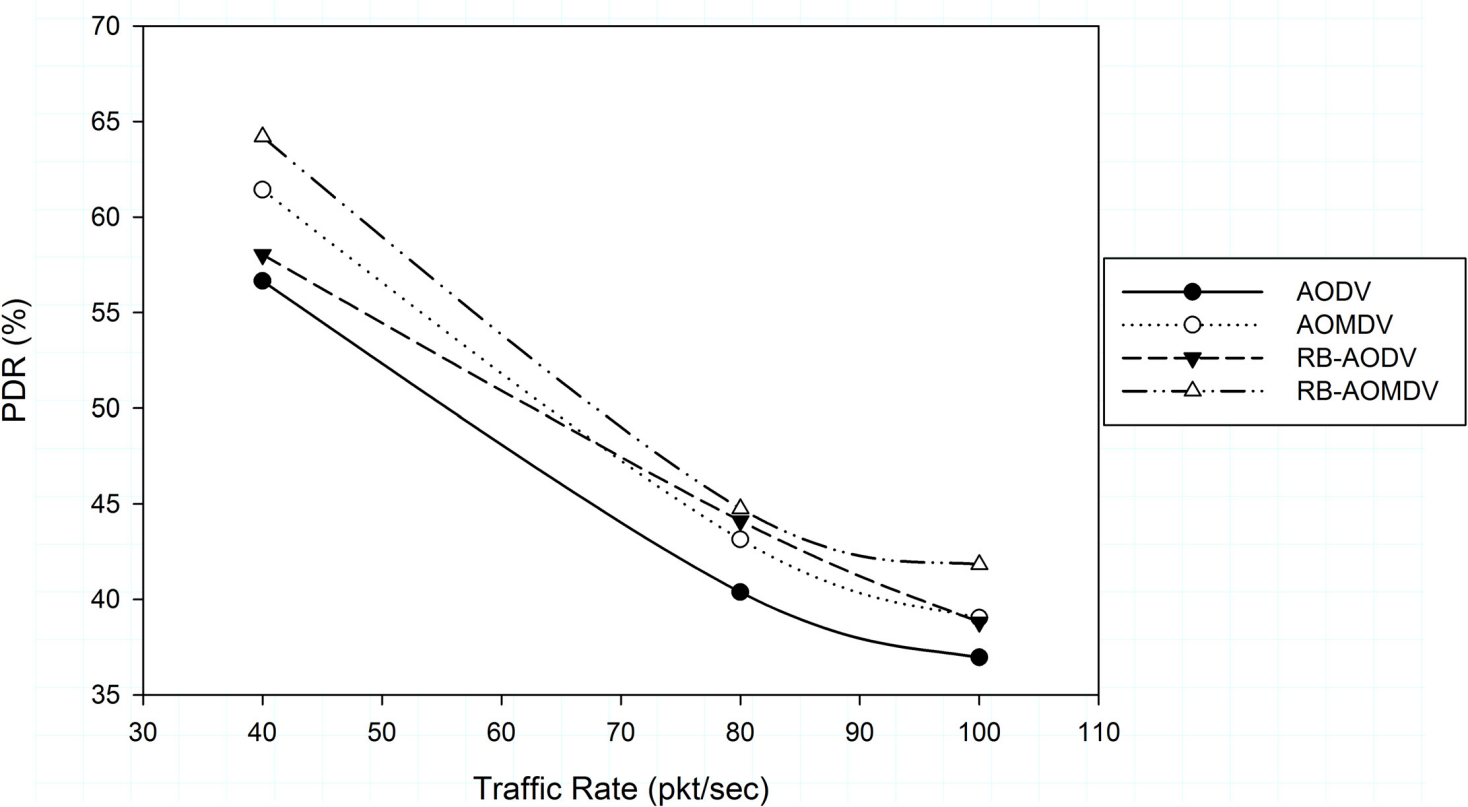
Packet delivery ratio with different traffic rate.

Fig 2 shows the effect of traffic load on the packet delivery ratio with different routing protocols. As can be seen, when traffic load increases the packet drop increases, which affects the throughput of the network. The RB-AOMDV protocol improves the reliability of the single version RB-AODV protocol by 11% and 8% at 40 pkt/sec and 100 pkt/sec respectively. Moreover, the RB-AOMDV protocol outperforms the AODV and AOMDV protocols by 13% and 7% at 100 pkt/sec respectively.

**Fig 3:**
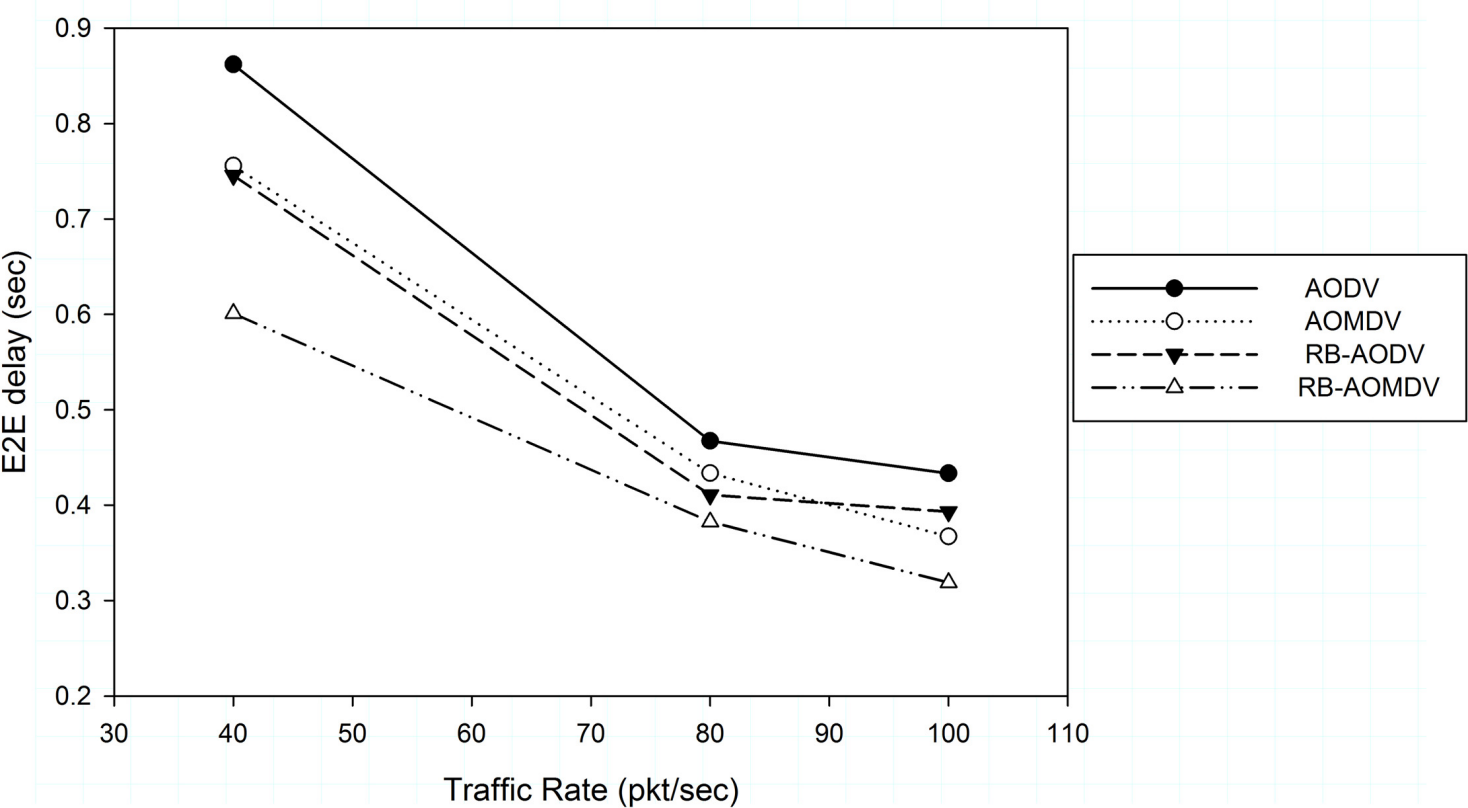
End to end delay with different traffic rate.

Fig 3 shows the results of average end to end delay in different traffic rates. Delay time in high traffic rate is low where the average packet time becomes shorter, but with high packet drop. Successfully transmitted packets take less time where more packets were buffered, although buffered packets will later be dropped as a result of high congestion. In this aggressive situation, the RB-AOMDV protocol shows better performance in terms of average delay. The lifetime of the packets to reach their destination is less than with the AOMDV protocol, with the advantage of control packets received from the receiver node. In addition, the use of multiple paths decreases the time needed to explore new paths when we compare that with the RB-AODV protocol. The improvements in moderated packet traffic rate (40 pkt/sec) are 19%, 20% and 30% less time delay compared with RB-AODV, AOMDV and AODV protocols respectively. In packet traffic rate (100 pkt/sec), the RB-AOMDV protocol shows less delay time with improvements of 18.8%, 13% and 26% compared with the RB-AODV, AOMDV and AODV routing protocols respectively. Adding the enhanced receiver node prediction of error in the network decreases the time needed to re-establish new updated multiple paths. As a result, the performance of delivery ratio is affected where the sender node uses alternative paths when receiving error packets and will update the routing table when receiving RRREQ packets from the receiver node.

**Fig 4:**
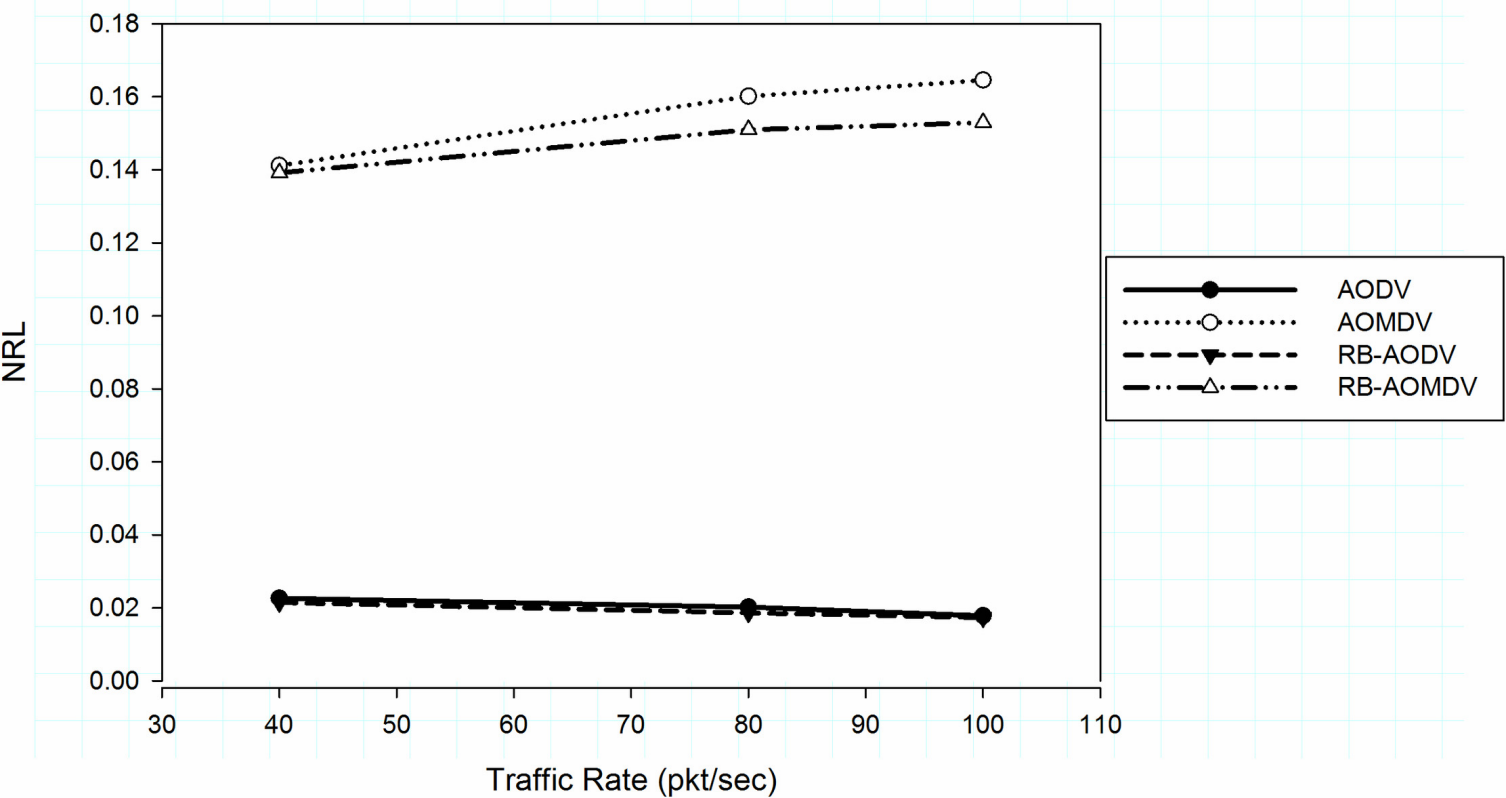
Normalized routing load with different traffic rate.

However, packet delivery ratio and average delay are improved and multipath protocols acquired more control packets, as depicted in Fig 4. The RB-AOMDV protocol increased the overhead compared with single path protocol in high traffic rate. The single path routing protocol gives better performance compared with multipath routing protocols in different packet rates. However, the RB-AOMDV protocol shows better results compared with the AOMDV protocol.

**Fig 5:**
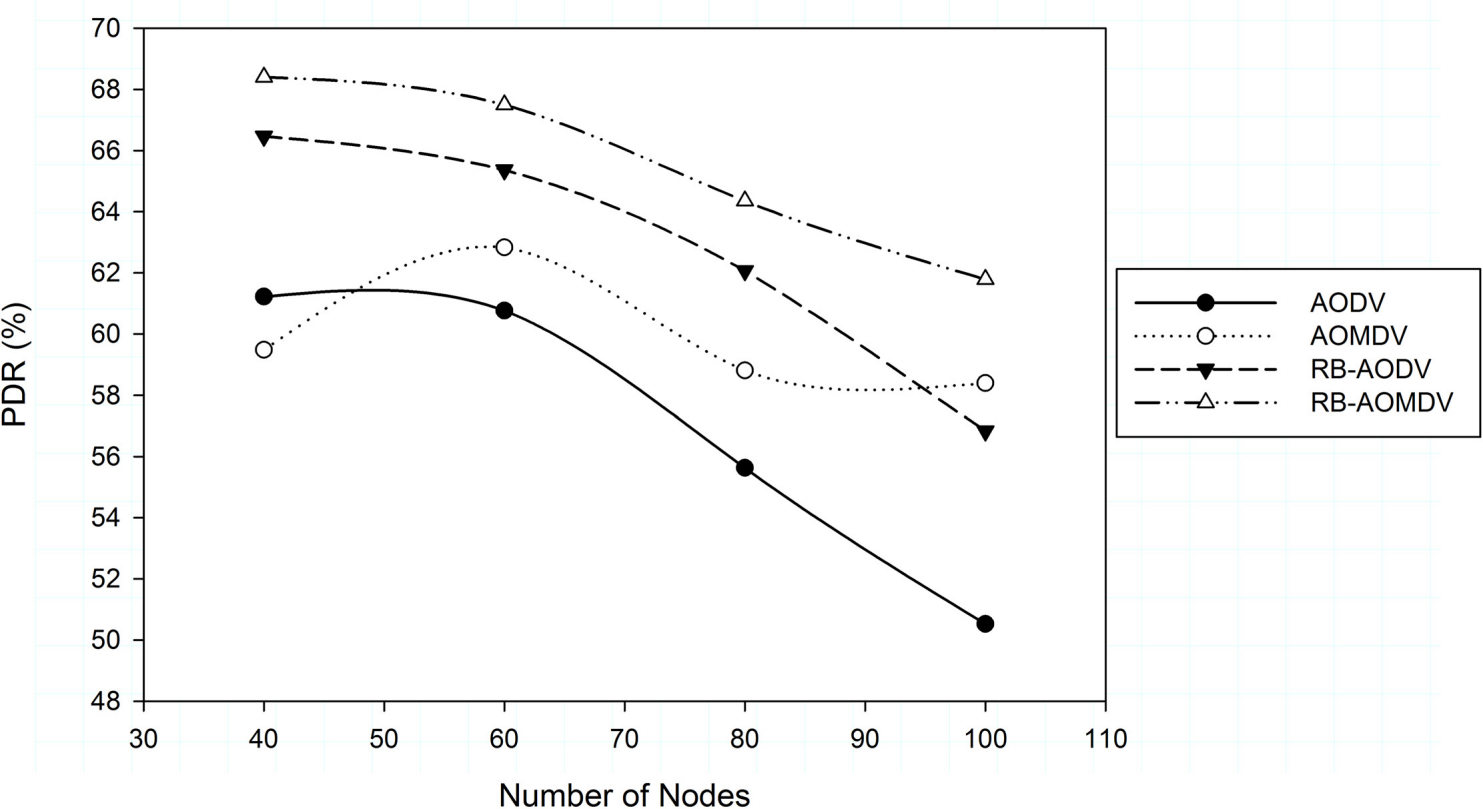
Packet delivery ratio with different number of nodes.

The next scenario measured the effect of node density on the protocol performance. Fig 5 shows the effect of node density on packet delivery ratio. It can be seen that when the number of nodes increases, the congestion increases as well. As a result, the data packet delivery ratio decreases. The RB-AOMDV protocol shows stable results compared with other protocols in all different numbers of nodes. When the number of nodes is 40, RB-AOMDV improved PDR by 15%, 11% and 3% compared with AOMDV, AODV and RB-AODV respectively. When the number of nodes increased, congestion increased, which affects the PDR. RB-AOMDV suffers less packet loss compared with other protocols; at 100 nodes the performance of PDR improved by 6%, 22% and 9% compared with AOMDV, AODV and RB-AODV routing protocols respectively.

**Fig 6:**
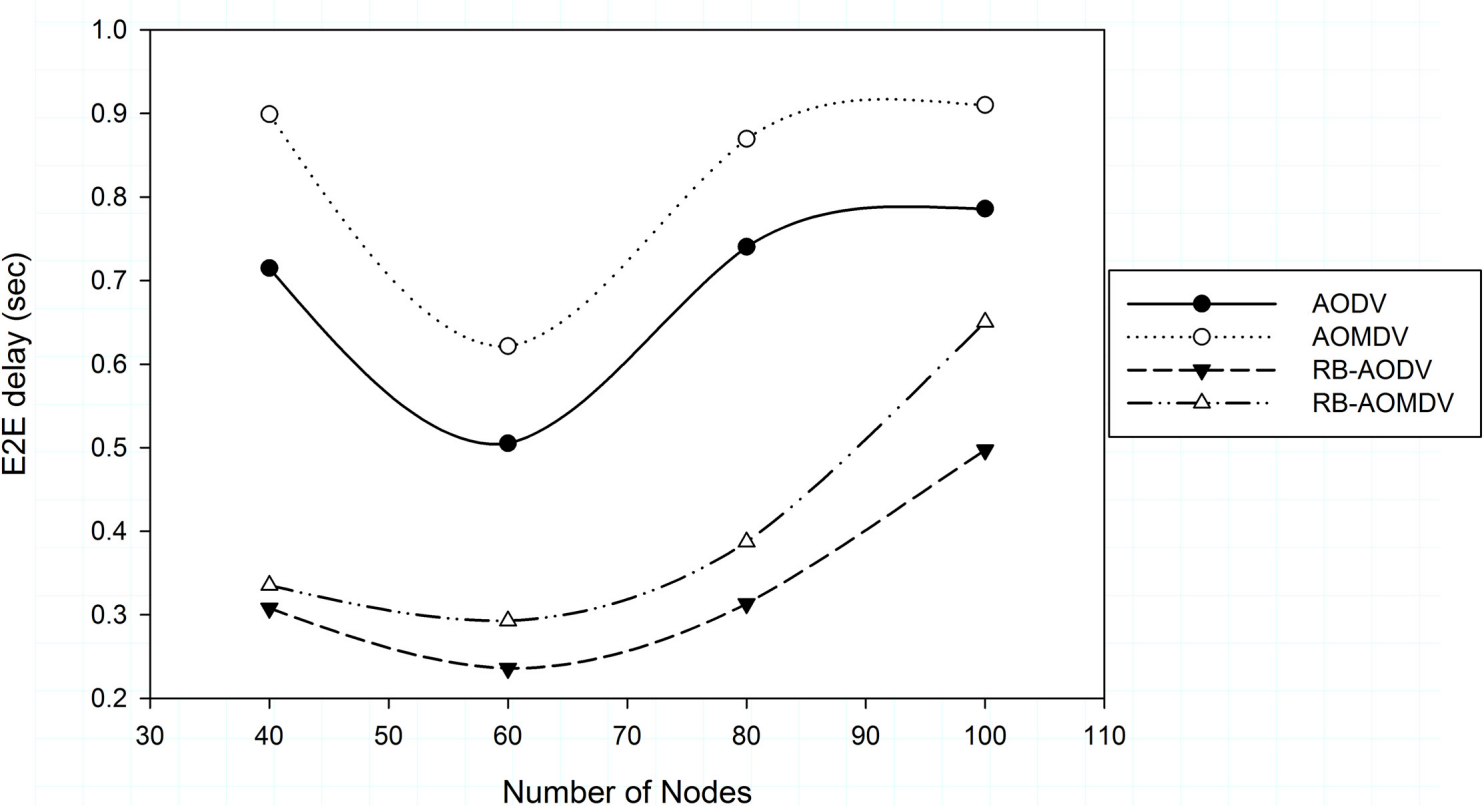
End to end delay with different number of nodes.

The average delay results in Fig 6 show that when the number of nodes is 60, the network connectivity is better where delay decreases. When the number of nodes is less, AODV and AOMDV protocols delay time increases with the increase of number of nodes. The multipath in high number of nodes and high mobility may result in using stale paths for forwarding data packets as alternative paths, which affects the network performance negatively more than in single path. Re-establishing new paths in single path when errors occur results in less time taken to use a valid path compared with the multipath version. The RB-AOMDV and RB-AODV protocols outperform the AODV and AOMDV protocols where time needed to set up new valid paths is less. RB-AOMDV outperforms AOMD by 62.71%, 52.88%, 55.49% and 28.53% less delay when the number of nodes is 40, 60, 80 and 100 respectively.

**Fig 7:**
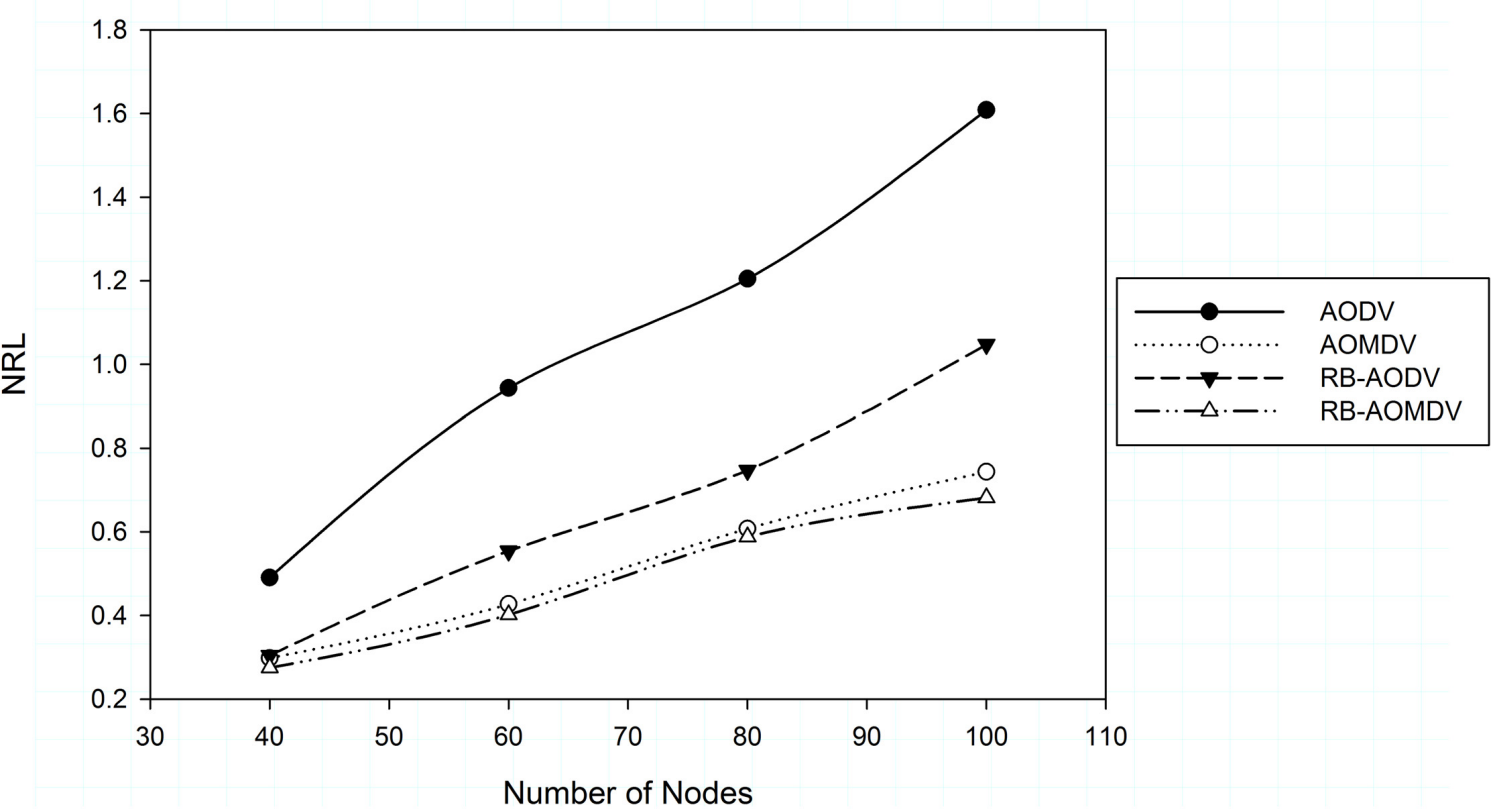
Normalized routing load with different number of nodes.

However, single path protocols take less time to reset-up new paths, and controls overhead performance is increased in high numbers of nodes, as shown in Fig 7. The increase in errors caused by congestion requires more broadcast of control packets over the network. In the multipath mechanism, the multiple paths explored decrease the need to re-establish new paths while the sender node select a new alternative path from the table to send data packets. In the RB-AOMDV protocol, while the receiver node is receiving data packets, the number of RRREQ packets being broadcast is less than with other routing protocols. The RB-AOMDV protocol outperforms other routing protocols in different numbers of nodes. The RB-AOMDV routing protocol improves NRL performance by 7.78% less when the number of nodes is 40, and 8.3% with 100 nodes compared with the AOMDV protocol.

## Conclusion

In this paper we proposed a RB-AOMDV routing protocol to decrease the time delay needed to re-establish a route discovery phase, especially when a network is predicted to be changed or congested. The receiver node keeps checking after a period of time whether data have been received or not. If received, it waits once again and then rechecks. If no data are received, the receiver node starts the discovery process by broadcasting the request packets. If after a number of trials no data are received, the receiver node stops the discovery process. Simulation results show that PDR and end-to-end delay outperform AODV, RB-AODV and AOMDV routing protocols in high traffic load and different numbers of nodes. Compared with AOMDV, RB-AOMDV improves the PDR by 7% and the end-to-end delay by 13% in high traffic load. With varied numbers of nodes, RB-AOMDV outperformed AOMDV with 15% PDR improvement and decreased delay by 62% when the number of nodes was 40. When the number of nodes went up to 100, RB-AOMDV improved PDR by 6% and decreased delay by 28%.

Selecting paths according to different QoS metrics constraints, or by using different optimization algorithms, as proposed in different single and multipath routing protocols in mobile ad hoc networks, is an open issue to be investigated using our proposed protocol.

## Supporting Information

S1 Appendix The simulation results of different runs with different traffic loads. (PDF)

S2 Appendix The simulation results of different runs with different number of nodes. (PDF)
