# Supplementary material for: Receiver-Based Ad Hoc On Demand Multipath Routing Protocol for Mobile Ad Hoc Networks
Source: PLoS One. 2016 Jun 3;11(6):e0156670. doi: 10.1371/journal.pone.0156670 (PMC4892561; doi:10.1371/journal.pone.0156670)

test under different runs for traffic load (40pkt/sec)

	E2E delay	NRL	PDR
AODV	0.800421	0.019	57.49817
	0.9117	0.03	44.40458
	0.829078	0.016	67.57221
	0.883856	0.024	57.164
	0.88437	0.024	56.656
Avg:	**0.861885**	**0.0226**	**56.65899**
AOMDV	0.664767	0.146	65.52936
	0.91021	0.131	47.3468
	0.618689	0.137	73.44572
	0.800244	0.149	61.936
	0.784451	0.143	58.862
Avg:	**0.755672**	**0.1412**	**61.42397**
RB-AODV	0.667215	0.02	55.63344
	0.738237	0.024	48.46572
	0.696881	0.021	71.72796
	0.754498	0.02	53.458
	0.871682	0.022	60.898
Avg:	**0.745703**	**0.0214**	**58.03662**
RB-AOMDV	0.650685	0.142	68.90195
	0.610491	0.127	51.2916
	0.589488	0.142	72.7295
	0.428855	0.142	67.258
	0.726476	0.143	60.822
Avg:	**0.601199**	**0.1392**	**64.20061**

test under different runs for traffic load (80 pkt/sec)

	E2E delay	NRL	PDR
AODV	0.468	0.016	44.058
	0.550319	0.021	23.038
	0.407631	0.007	62.352
	0.402656	0.029	31.128
	0.508357	0.028	41.286
Avg:	**0.467393**	**0.0202**	**40.3724**
AOMDV	0.348239	0.164	48.03
	0.498397	0.154	25.728
	0.360393	0.153	64.028
	0.511962	0.167	32.904
	0.448698	0.163	44.964
Avg:	**0.433538**	**0.1602**	**43.1308**
RB-AODV	0.357407	0.027	48.55
	0.316656	0.018	31.512
	0.483466	0.013	56.418
	0.38328	0.016	42.444
	0.512514	0.019	41.604
Avg:	**0.410665**	**0.0186**	**44.1056**
RB-AOMDV	0.347315	0.154	49.51
	0.397962	0.151	25.35
	0.329351	0.15	64.472
	0.412426	0.152	39.528
	0.425689	0.159	44.868
Avg:	**0.382549**	**0.1532**	**44.7456**

test under different runs for traffic load (100 pkt/sec)

		E2E delay	NRL	PDR
AODV		0.405152	0.017	46.712
		0.428385	0.027	18.45
		0.40139	0.011	54.57
		0.460621	0.018	26.146
		0.471271	0.016	38.882
	Avg:	**0.433364**	**0.0178**	**36.952**
AOMDV		0.305768	0.164	46.622
		0.445433	0.148	21.958
		0.302994	0.162	58.574
		0.402691	0.179	26.018
		0.378777	0.17	41.978
	Avg:	**0.367133**	**0.1646**	**39.03**
RB-AODV		0.349222	0.018	47.362
		0.332742	0.026	24.034
		0.400624	0.009	56.412
		0.374259	0.019	30.244
		0.508412	0.014	35.982
	Avg:	**0.393052**	**0.0172**	**38.8068**
RB-AOMDV		0.309228	0.153	49.522
		0.269166	0.155	25.38
		0.254132	0.154	61.636
		0.387671	0.155	29.416
		0.374622	0.152	43.16
	Avg:	**0.318964**	**0.1538**	**41.8228**

Avg tests under different traffic load (pkt/sec)

E2EDAELAY

Column1	40	80	100
AODV	0.861885	0.467393	0.433364
AOMDV	0.755672	0.433538	0.367133
RB-AODV	0.745703	0.410665	0.393052
RB-AOMDV	0.601199	0.382549	0.318964

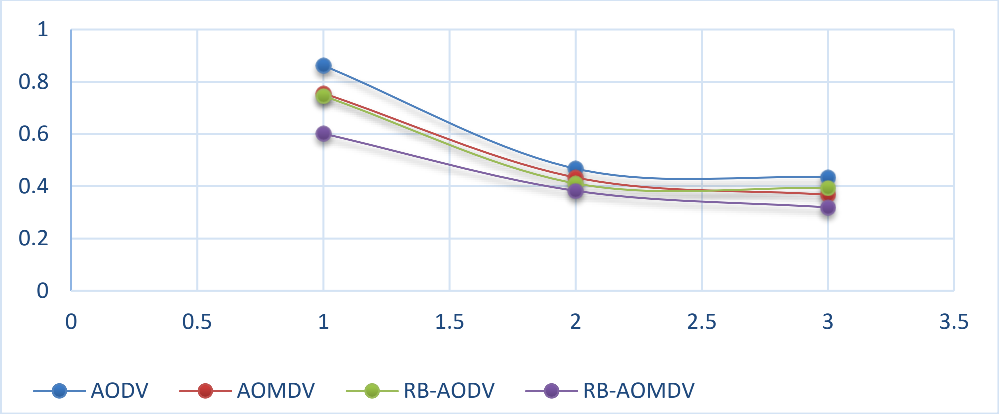

NRL

Column1	40	80	100
AODV	0.0226	0.0202	0.0178
AOMDV	0.1412	0.1602	0.1646
RB-AODV	0.0214	0.0186	0.0173
RB-AOMDV	0.1392	0.1532	0.1538

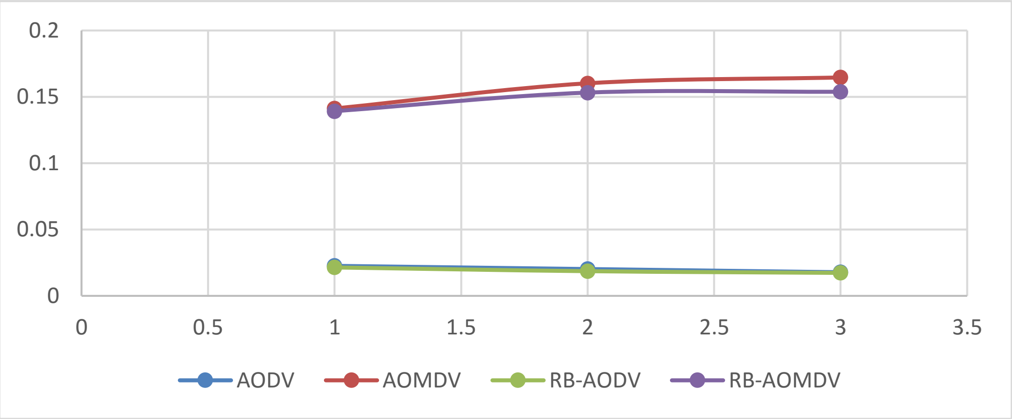

PDR

Column1	40	80	100
AODV	56.658991	40.3724	36.952
AOMDV	61.423975	43.1308	39.03
RB-AODV	58.036623	44.1056	38.8068
RB-AOMDV	64.20061	44.7456	41.8228

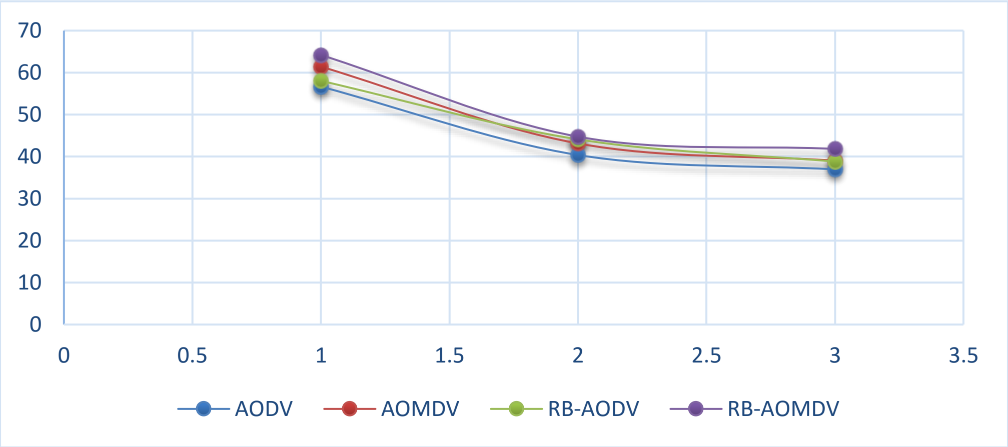

Supplement: S1 Appendix — (PDF) [file pone.0156670.s001.pdf]
