# Supplementary material for: Receiver-Based Ad Hoc On Demand Multipath Routing Protocol for Mobile Ad Hoc Networks
Source: PLoS One. 2016 Jun 3;11(6):e0156670. doi: 10.1371/journal.pone.0156670 (PMC4892561; doi:10.1371/journal.pone.0156670)

test under different runs for number of nodes 40 nodes

	E2E delay	NRL	PDR
AODV	0.854566	0.495	54.71394
	0.499951	0.628	54.81267
	0.778212	0.353	68.76149
	0.779925	0.448	65.91449
	0.661871	0.529	61.89585
Avg:	**0.714905**	**0.4906**	**61.21969**
AOMDV	0.940131	0.324	55.93376
	1.089989	0.296	54.06889
	0.752941	0.3	66.80916
	0.917281	0.283	60.34231
	0.793906	0.288	60.25426
	**0.89885**	**0.2982**	**59.48168**
RB-AODV	0.375493	0.269	61.40335
	0.210172	0.395	61.15691
	0.182305	0.184	74.98328
	0.383128	0.333	68.56265
	0.387259	0.342	66.21988
Avg:	**0.307671**	**0.3046**	**66.46521**
RB-AOMDV	0.577123	0.262	59.12382
	0.335084	0.248	59.20778
	0.266677	0.271	70.03774
	0.403863	0.254	69.33148
	0.093158	0.34	84.2917
Avg:	**0.335181**	**0.275**	**68.3985**

test under different runs for number of nodes 60 nodes

	E2E delay	NRL	PDR
AODV	0.425828	1.149	53.47301
	0.598887	0.814	64.86545
	0.570589	0.679	68.69348
	0.354604	0.837	69.18355
	0.575318	1.238	47.61367
Avg:	**0.505045**	**0.9434**	**60.76583**
AOMDV	0.798832	0.431	60.0329
	0.583764	0.387	73.73264
	0.627759	0.435	70.58843
	0.335331	0.386	80.11867
	0.761196	0.495	54.69646
Avg:	**0.621376**	**0.4268**	**67.83382**
RB-AODV	0.272079	0.717	60.48811
	0.273399	0.53	70.55772
	0.240992	0.351	69.14103
	0.087259	0.401	76.17844
	0.30525	0.772	50.45283
Avg:	**0.235796**	**0.5542**	**65.36362**
RB-AOMDV	0.511314	0.386	62.39138
	0.245799	0.384	75.79625
	0.156598	0.423	67.03759
	0.254881	0.402	77.95449
	0.295401	0.414	54.31019
Avg:	**0.292799**	**0.4018**	**67.49798**

test under different runs for number of nodes 80 nodes

	E2E delay	NRL	PDR
AODV	0.941641	1.165	48.53113
	0.490645	0.825	71.5848
	0.5216	1.841	47.86216
	0.737988	1.237	56.55556
	1.009154	0.954	53.60966
Avg:	**0.740206**	**1.2044**	**55.62866**
AOMDV	1.179862	0.651	50.90876
	0.412678	0.551	71.31465
	0.772652	0.563	59.64801
	0.98368	0.583	59.14111
	0.997784	0.69	53.03523
Avg:	**0.869331**	**0.6076**	**58.80955**
RB-AODV	0.423735	0.646	59.20015
	0.199124	0.514	67.50362
	0.260428	1.436	52.71287
	0.302584	0.584	71.81563
	0.380314	0.555	59.10998
Avg:	**0.313237**	**0.747**	**62.06845**
RB-AOMDV	0.407254	0.545	54.79093
	0.340855	0.532	73.37458
	0.440205	0.571	59.93664
	0.438961	0.55	63.39026
	0.307584	0.742	70.26781
Avg:	**0.386972**	**0.588**	**64.35204**

test under different runs for number of nodes 100 nodes

	E2E delay	NRL	PDR
AODV	0.77543	1.79	47.69486
	0.812246	1.673	45.97975
	0.838683	1.156	59.80726
	0.677284	1.471	57.37222
	0.826074	1.951	41.7644
Avg:	**0.785943**	**1.6082**	**50.5237**
AOMDV	0.91316	0.706	58.2408
	1.021274	0.815	51.88938
	0.960776	0.786	60.58145
	0.787376	0.693	64.33012
	0.866748	0.717	56.90971
Avg:	**0.909867**	**0.7434**	**58.39029**
RB-AODV	0.433681	1.295	51.90184
	0.576192	0.984	54.21408
	0.63271	0.702	63.226
	0.40069	1.068	61.38098
	0.441735	1.189	53.43866
Avg:	**0.497002**	**1.0476**	**56.83231**
RB-AOMDV	0.775941	0.664	61.21172
	0.61735	0.679	57.04725
	0.717563	0.705	67.54883
	0.609411	0.699	64.68777
	0.53125	0.661	58.43319
Avg:	**0.650303**	**0.6816**	**61.78575**

Avg tests under different Number of Nodes

e2edelay

	40	60	80	100
AODV	0.714905	0.505045	0.740206	0.785943
AOMDV	0.89885	0.621376	0.869331	0.909867
RB-AODV	0.307671	0.235796	0.313237	0.497002
RB-AOMDV	0.335181	0.292799	0.386972	0.650303

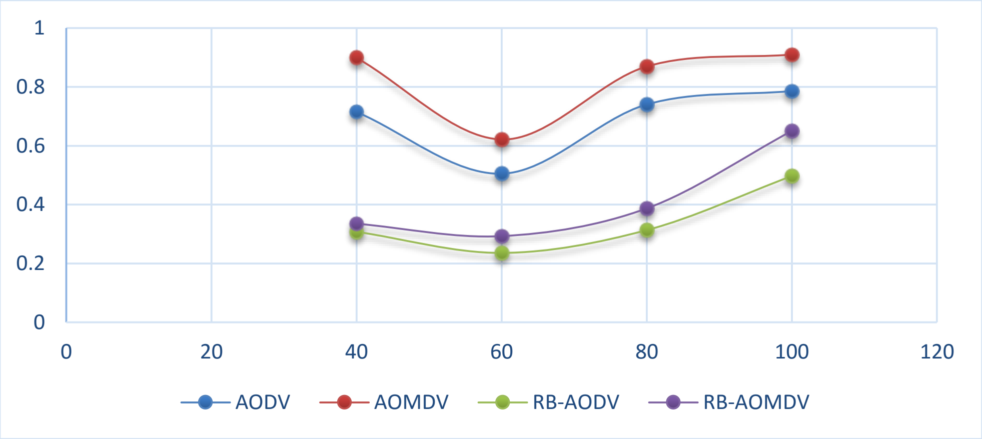

NRL

	40	60	80	100
AODV	0.4906	0.9434	1.2044	1.6082
AOMDV	0.2982	0.4268	0.6076	0.7434
RB-AODV	0.3046	0.5542	0.747	1.0476
RB-AOMDV	0.275	0.4018	0.588	0.6816

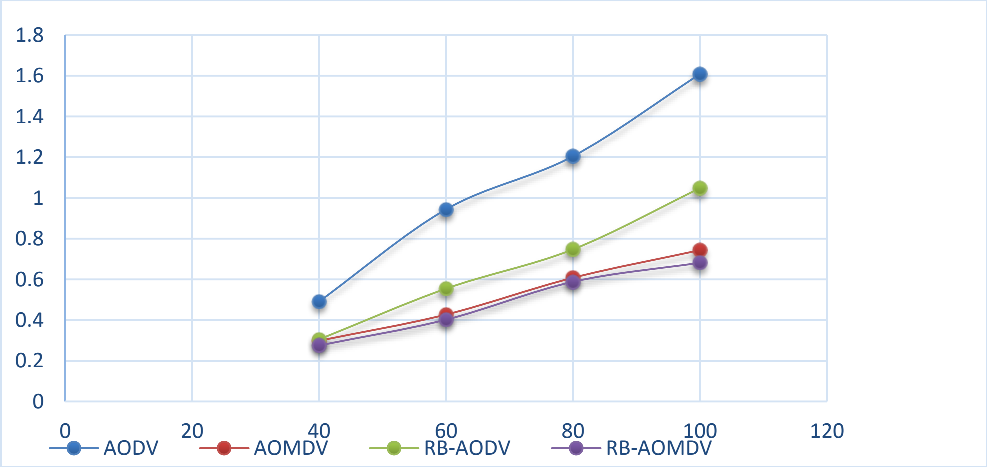

PDR

	40	60	80	100
AODV	61.21969	60.76583	55.62866	50.5237
AOMDV	59.48168	62.83382	58.80955	58.39029
RB-AODV	66.46521	65.36362	62.06845	56.83231
RB-AOMDV	68.3985	67.49798	64.35204	61.78575

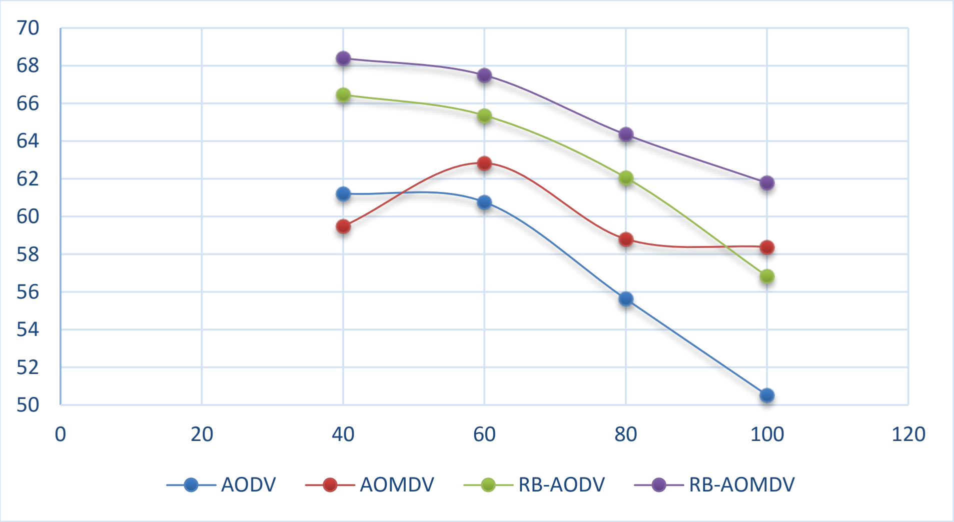

Supplement: S2 Appendix — (PDF) [file pone.0156670.s002.pdf]
